# An optimal position for elbow arthrodesis to accommodate modern daily living activities

**DOI:** 10.3325/cmj.2025.66.273

**Published:** 2025-08

**Authors:** Ivo Buhač, Damjan Dimnjaković, Igor Knežević, Ivan Bojanić

**Affiliations:** 1 Health Center Široki Brijeg, Široki Brijeg, Bosnia and Herzegovina; 2 Department of Orthopaedic Surgery, Zagreb University Hospital Center, Zagreb, Croatia; 3 University of Zagreb, School of Medicine, Zagreb, Croatia

## Abstract

**Aim:**

To assess the optimal fusion angle of the elbow to accommodate the activities of daily living.

**Methods:**

The study enrolled 30 healthy adult volunteers (mean age 24 years), who performed 29 activities with an elbow brace fixed at various flexion angles (30°, 50°, 70°, 90°, 110°, 120°). The activities were divided into three groups: the activities of daily living, personal care and hygiene, and modern activities of daily living. Activity performance was scored on a scale previously designed to assess the difficulty of performing a particular activity.

**Results:**

The highest overall score for all activities was at 90° elbow flexion (79.70 ± 4.11), followed by 110° (76.17 ± 4.01) and 70° (75.43 ± 6.64). For modern activities of daily living, the highest score (43.77 ± 1.33) was also at 90°. The 90° angle also allowed the highest percentage (86.6%) of task completion in all activity groups.

**Conclusion:**

Our study supports making elbow arthrodesis at 90° of flexion, which may also be applied to modern daily living activities. However, patients should be consulted about the preferred angle for elbow arthrodesis, as no single fusion angle allows for the performance of all modern daily living activities.

Elbow arthrodesis is a surgical procedure that was historically used for the treatment of elbow tuberculosis (1). Nowadays, indications have expanded to secondary posttraumatic osteoarthritis, severe elbow instability, massive comminuted intra-articular fractures of the distal humerus, nonunion and malunion fractures around the elbow and neuropathic joint, as well as war-related injuries and elbow infections (1-5). Additionally, it may be an alternative to total elbow arthroplasty in younger patients and a salvage procedure after a failed elbow arthroplasty (4,6,7).

Unlike knee or ankle arthrodesis, elbow arthrodesis represents a considerable functional deficit for an individual (4,6,8). This is primarily due to the inability of the adjacent shoulder and wrist to compensate for the lack of movement in the elbow. Therefore, determining an optimal angle for elbow fusion is still being debated. These findings are based on anecdotal reports and only a few functional studies (3,9-13). Some suggested that 90° of elbow flexion is the standard for unilateral elbow arthrodesis (1,6). However, other studies recommended a variety of angles, from those closer to extension to the ones greater than 90° of flexion (1,14,15). Apart from the fusion angle and the compensatory mechanisms, the function after elbow arthrodesis may also be altered by the quality of bone, which determines surgical success and postoperative adaptations.

In 1981, Morrey et al investigated the ability to perform different activities of daily living (ADL) and personal care hygiene (PCH) in various elbow positions (3). Later studies mostly selected the activities for testing based on this research, suggesting the ability to successfully perform activities in different positions (8-10,14,16,17). In 2001, Tang et al proposed a classification system scoring the ability to perform a specific activity considering elbow motion restrictions (14). Another pivotal point was the development of technologies that changed the ADL. In the study by Morrey et al, ADL were based on feeding, drinking, standing up from a chair, opening doors, using a phone, and reading newspapers (3). Nowadays, ADL are vastly determined by the use of electronic devices, mainly the computer, smartphone, tablet, and other modern gadgets (18). Only a few studies have investigated functional elbow positions when using some of these devices (16,19). Still, none of them have determined the optimal elbow position for arthrodesis concerning these activities. In 2018, Oosterwijk et al reviewed the literature on the shoulder and elbow range of motion while performing different activities (17). The only activities that might be considered modern were using the cellular phone, keyboard, and mouse. The aim of this study was to determine the optimal fusion angle of the elbow with regard to ADL and PCH, as well as modern activities of daily living (mADL).

## PATIENTS AND METHODS

The study enrolled thirty adult volunteers between February and April 2024, who were all students from the University of Zagreb. A sample size of convenience was used. All volunteers were healthy, and anyone with impairments, previous injuries, or surgeries of the upper extremities was not included. The volunteers signed an informed consent at the beginning of the study, which was approved by the University of Zagreb Institutional Review Board and the Zagreb University Hospital Centre Review Board. Demographic data, including the volunteer’s age, sex, height, and body mass, were noted.

**Figure 1:**
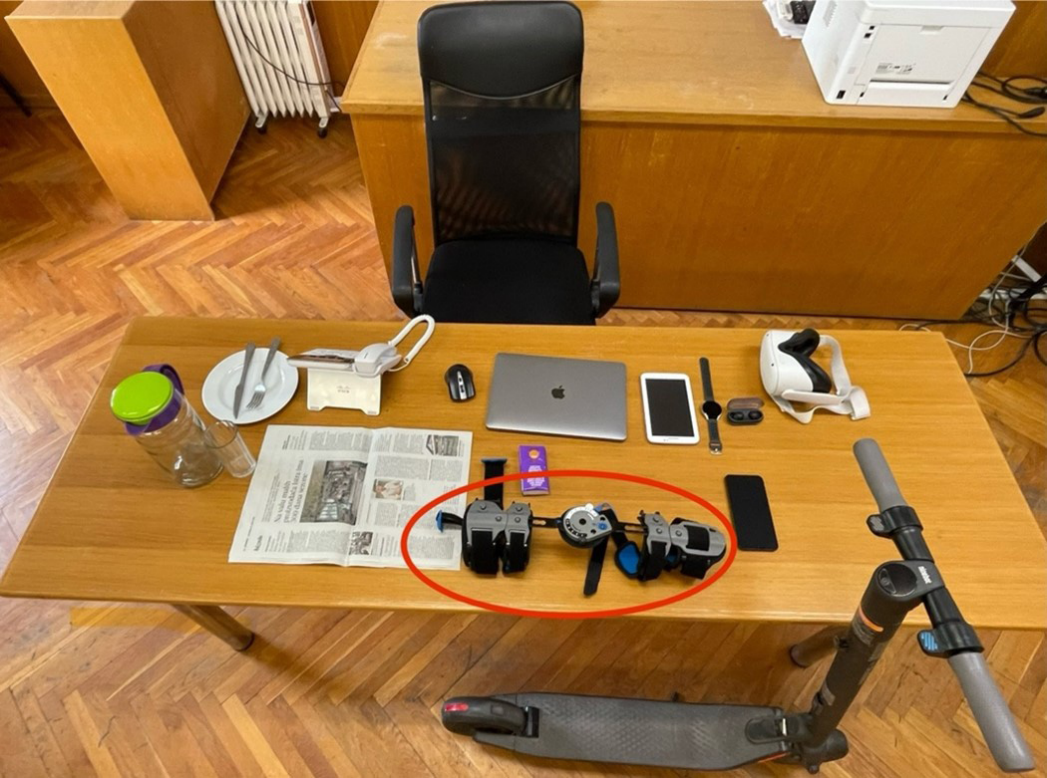
Equipment used in all the activities – elbow brace (marked in red), chair with backrest, water pitcher, water glass, cutlery (plate, knife, fork), daily newspaper, paper tissues, landline telephone, computer mouse, laptop, tablet, smartwatch, wireless earbuds, smartphone, virtual and augmented reality headset, and electric scooter.

**Figure 2:**
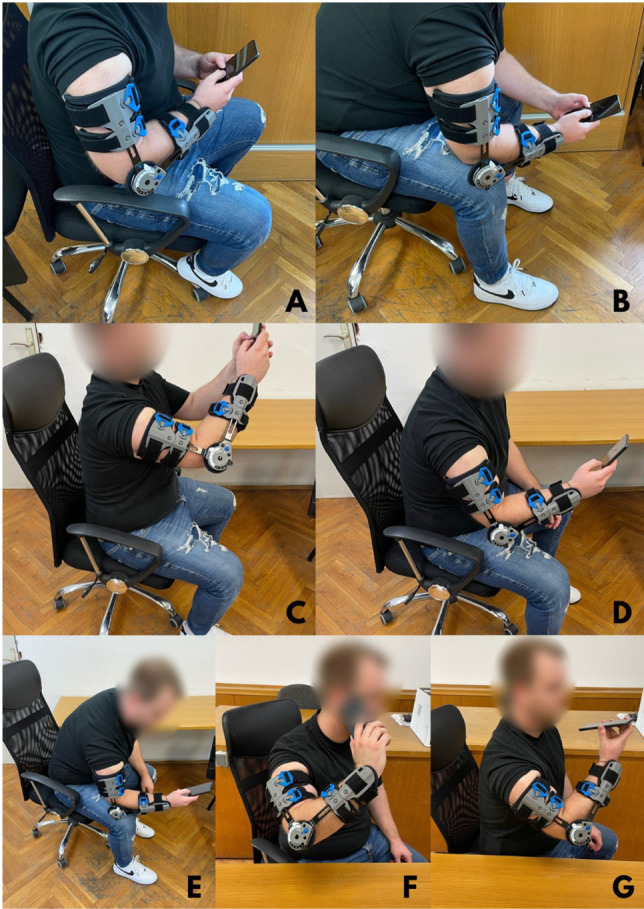
Assessment of modern daily activities – using a smartphone in different positions. **(A)** Holding a smartphone with both hands at chest level and typing with both thumbs; **(B)** holding a smartphone with both hands with the trunk leaning forward and the elbows resting on the thights while typing with both thumbs; **(C)** holding a smartphone with both hands at eye level and typing with both thumbs; **(D)** holding a smartphone with the dominant hand and typing with the thumb; **(E)** scrolling for news using a smartphone; **(F)** placing a smartphone to the ipsilateral ear for making a phone call; **(G)** placing a smartphone in front of the mouth for making a phone call.

**Figure 3:**
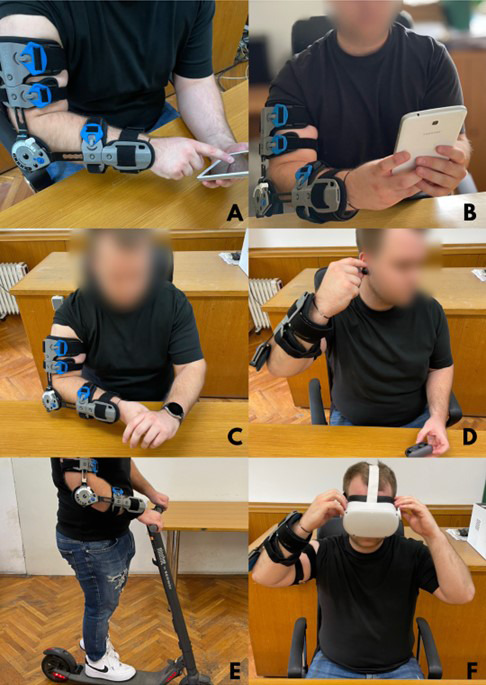
Assessment of modern daily activities. **(A)** Using a tablet for text typing; **(B)** using a tablet for gaming; **(C)** adjusting a smartwatch on the contralateral wrist; **(D)** placing a wireless earbud in the ipsilateral ear; **(E)** standing on an electric scooter; **(F)** adjusting a virtual and augmented reality headset.

The examination was performed only on the dominant arm. To ensure that volunteers performed the same activity in the same manner, a single examiner instructed each volunteer to perform the activities. The activities of ADL and PCH were the same as described by Morrey et al, apart from combing the hair, which was defined as a single activity instead of two separate activities of touching the vertex and the occiput of the head (3). The equipment used in all activities is shown in Figure 1, while the equipment specifically used for mADL (Table 1) is displayed in Figures 2 and 3. Each of the activities was performed with the elbow in a different position, using a hinged elbow brace (Rebound® Post-Op Elbow, Össur, Reykjavik, Iceland) to constrain the elbow to a desired flexion angle. The brace was placed on the volunteer’s arm according to the manufacturer's instructions and adjusted to fit the elbow comfortably. All activities were initially performed without the brace. Next, the activities were attempted with the fitted brace secured at 30°, 50°, 70°, 90°, 110°, and 120° of elbow flexion. A single examiner noted each activity in each elbow position based on the volunteer’s impression using the classification system by Tang et al (14). This classification system uses grades from 0 to 3 points, with the highest being the best possible score, representing no difficulties in performing the activity.

**Table 1:** The tested modern activities of daily living

	Activity
1	Using a laptop keyboard
2	Using a computer mouse
3	Holding a smartphone with both hands at chest level and typing with both thumbs
4	Holding a smartphone with both hands while leaning forward and the elbows resting on the tights while typing with both thumbs
5	Holding a smartphone with both hands at eye level and typing with both thumbs
6	Holding a smartphone with the dominant hand and typing with thumb
7	Scrolling for news using a smartphone
8	Placing a smartphone to the ipsilateral ear for making a phone call
9	Placing a smartphone to the mouth for making a phone call
10	Using a tablet for text typing
11	Using a tablet for gaming
12	Adjusting a smartwatch on the contralateral wrist
13	Placing wireless earbuds in the ipsilateral ear
14	Standing on an electric scooter
15	Adjusting a virtual and augmented reality headset

### Statistical analysis

The normality of distribution was assessed by reviewing skewness and kurtosis. A one-way repeated-measures ANOVA with Bonferroni adjustment was conducted to assess the differences in the mean functional score between different elbow flexion positions (30°, 50°, 70°, 90°, 110°, 120°) during ADL, mADL, and PCH. The scores between different elbow positions for dependent variables were compared using the *t* test. The level of significance was set at *P* < 0.05. Statistical analysis was performed with SPSS, version 30, (IBM Corp, Armonk, NY, USA).

## RESULTS

The study enrolled 30 healthy volunteers (12 female) with a mean age of 24 (range, 19-26) years. In all volunteers, the right hand was the dominant hand. The average body mass index was 24.6 (range, 18.8-32.2) kg/m^2^.

The highest average score (79.70 ± 4.11) for all activities combined was found at 90° of elbow flexion, followed by 110° (76.17 ± 4.01) and 70° (75.43 ± 6.64). The exact trend can be observed in ADL, mADL, and combined ADL and mADL groups. In the PCH group, the highest mean score (14.07 ± 1.84) was also noted at 90° of elbow flexion, followed by 70° (13.70 ± 2.10) and 110° (12.50 ± 1.76). The scores in all activity groups were significantly higher at 90° than at 110° (*P* < 0.001, *t* test) (Table 2).

**Table 2:** The mean scores of the volunteer’s activity performance at different degrees of elbow flexion for each activity group*

Degree of elbow flexion	ADL^†^ (24.00)^§^	mADL^†^ (45.00)^§^	ADL + mADL^†^ (69.00)^§^	PCH^†^ (18.00)^§^	ADL + mADL + PCH^†^ (87.00)^§^
30	14.80 (1.32)	32.30 (2.67)	47.10 (3.74)	9 (6-18)^‡^	56.10 (5.32)
50	16.70 (2.33)	36.63 (3.16)	53.60 (5.25)	10.40 (1.71)	64.00 (6.76)
70	20.53 (2.43)	41.20 (2.68)	61.73 (4.83)	13.70 (2.10)	75.43 (6.64)
90	21.87 (1.79)	43.77 (1.33)	65.63 (2.76)	14.07 (1.84)	79.70 (4.11)
110	21.17 (1.51)	42.50 (1.57)	63.67 (2.78)	12.50 (1.76)	76.17 (4.01)
120	19.03 (2.53)	40.47 (2.45)	59.50 (4.63)	10.53 (1.59)	70.03 (5.54)

*Abbreviations: ADL – activities of daily living; mADL – modern activities of daily living; PCH – personal care and hygiene activities.

†Data are expressed as a mean (standard deviation).

‡Data are expressed as a median (range).

§Maximum possible score for each activity group.

**Figure 4:**
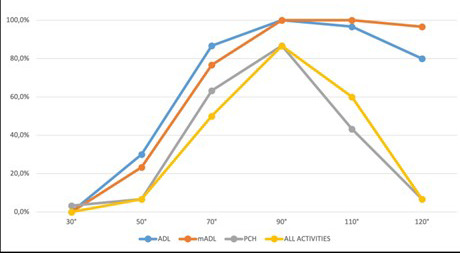
The proportion of volunteers capable of completing all tasks within each category of activities. ADL – activities of daily living; mADL – modern activities of daily living; PCH – personal care and hygiene activities.

**Figure 5:**
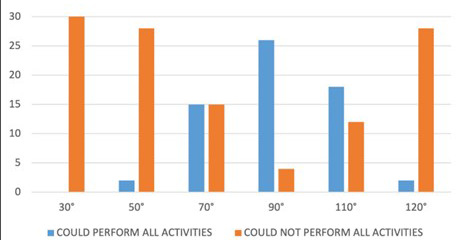
The number of volunteers based on their capability to complete all activities, contingent upon elbow flexion angle constraints.

The rate of successful activity completion depending on the elbow flexion degree is presented in Figure 4. Constraining the elbow in 90° of flexion allowed 86.6% (26 of 30) of the volunteers to complete all the activities successfully (Figure 5); four participants did not manage to touch the sacrum. At 110° of elbow flexion, 60% of volunteers could perform all the activities, while 50% could do so at 70° elbow flexion. At 50° or 120° of flexion, only two (6.6%) volunteers could complete all the activities. None of the volunteers could complete all the activities at 30° of flexion.

## DISCUSSION

Our results align with those of previous studies (2,9,10), suggesting 90° elbow flexion as the most preferable position when performing elbow arthrodesis. These results were confirmed for all the examined activity groups. To our knowledge, this is the first study to determine the optimal position for elbow arthrodesis regarding modern activities of daily living.

Most previous studies have tried to determine the necessary range of motion of the elbow for certain activities (3,10,16,17). This study is one of the few investigating how easily volunteers can perform certain activities with a fixed elbow position. The basis for most of the studies regarding elbow range of motion is the study by Morrey et al performed on 33 healthy individuals aged 21 to 75 (3). The authors used the triaxial electrogoniometer to determine the ranges of motion needed to perform eight ADL and seven PCH activities. They defined the functional arc of elbow flexion/extension from 30° to 130°. Although the study, published in 1981, was the cornerstone for further similar studies, due to technological development, ADL have changed over time (3,18). This was why the number of everyday activities involving gadgets increased and became a point of further interest. Nagy et al expanded the list of activities by including typing on a keyboard in 1999, and Sardelli et al in 2011 added the use of the computer mouse and the mobile phone, which was, at that time, only a feature phone and not a smartphone (9,16). Sardelli et al enrolled 25 healthy volunteers. They examined the functional elbow range of motion during most of the activities described by Morrey et al using a ten-camera, optical, three-dimensional motion analyzer. The results suggested the functional range of motion in the elbow to be greater than that reported by Morrey et al, with an arc between 27°±7° and 149°±5°. The greatest elbow flexion angle was needed for mobile phone (147°±3°) and stationary phone use (146°±3°) (16). Our study further expanded the number of modern activities arbitrarily determined and described as mADL. These activities include the use of smartphones, tablets, wireless headphones, smartwatches, electric scooters, and augmented/virtual reality goggles. Our study suggests that the angle of 110° (20.76 points) elbow flexion is the preferable position for seven activities related to smartphone use. This was followed by 90° (20.60 points) and 120° (20.06 points) elbow flexion. This again shows the differences that might result from using the smartphone compared with the feature phone since Sardelli et al only examined a single activity, ie, picking up the phone and placing it at the ipsilateral ear (16). Moreover, we assessed the elbow position while using four different styles of typing on smartphones as suggested by Ko et al (19) and adding the positions to read the news, placing the phone on the ipsilateral ear, and placing the phone in front of the mouth for conversation.

Since the elbow is one of the joints positioning the hand in space, a lack of elbow motion presents a great functional deficit, limiting ADL (4,5). O’Neill et al have shown that, contrary to the general belief, the shoulder does not significantly compensate for a lack of elbow motion (8). These facts and the diversity of elbow flexion angles needed for different activities explain why there is no consensus on the optimal position for unilateral elbow arthrodesis (4-6,9,14,20). Nevertheless, our study and many others have suggested that 90° of flexion is the best position for elbow arthrodesis (2,9,10). We used a hinged elbow brace and alternated the fixed position of elbow flexion from 30° to 120° to examine different activities in our volunteers. One of the studies using a hinged elbow brace to explore the ability to perform 12 elbow activities was performed by Vasen et al in 1995 (10). The study was conducted on 50 healthy volunteers with a mean age of 36 years. In contrast to our study, their focus was allowing a specific range of motion between 30° and 135° of flexion. First, the flexion would be subsequently limited from 135° by increments of 15° until reaching 30°. Second, the brace was reset to initial elbow movement restriction from 30° to 135° of flexion, followed by subsequent extension limitations from 30° incrementally by 15° until reaching 90°. Finally, with the starting position between 90° and 135°, the flexion was subsequently limited by increments of 15° until reaching 90°. The subject and the examiner determined whether the subject could perform a required activity. With the elbow fixed at 90°, 93% of volunteers could feed themselves, but 87% could not touch their necks. In our study, at 90° of elbow flexion, 86.7% of volunteers could perform all activities. The worst score was observed at 30°, when none of the volunteers could perform all activities (Figure 5). Vasen et al also observed that at 30° elbow flexion, 98% of volunteers could not drink and 94% could not feed themselves (10).

Nagy et al also favored the 90° angle of flexion for elbow arthrodesis (9). The study was conducted on 26 healthy volunteers aged 20 to 60 and consisted of two phases. In the first phase, both elbows were immobilized with a hinged elbow brace at 45° and then at 90° flexion. The volunteers had to grade the performance of 25 activities as “easy,” “awkward,” “difficult,” or “impossible.” At 90°, over 80% of volunteers graded all the activities as “easy” or “awkward.” In comparison, at 45°, 90% of volunteers graded several activities as “impossible,” including touching the occiput, mouth, and opposite shoulder, drinking from a glass and using a phone. In the second phase, the volunteers had their elbows immobilized with a cast. They were randomized into a group with immobilization of the non-dominant arm or a group with immobilization of the dominant arm. The elbow was immobilized for 24 hours, first in 45° and later in 90° flexion, with a period between immobilizations of 2 to 7 days. After the final cast removal follow-up, the volunteers were asked to choose the preferred of the two positions for eight specific activities. In 88% of volunteers, the preferred position was 90° flexion.

Since the mere ability to perform an activity is sometimes difficult to determine and depends on compensatory movements, we used a scoring system introduced by Tang et al to quantify the difficulty of performing a particular activity (14). The authors assessed 24 healthy volunteers while performing different activities as described by Morrey et al in ADL and PCH groups, with a hinged elbow brace fixed in 30°, 50°, 70°, 90°, 110° and 130° flexion (3,14). Unlike ours, their study showed the best results at 110° flexion, with a significantly higher score compared with the 90° of flexion in the ADL and all activity groups. They also found the best results at 110° of flexion in the PCH group, but there was no significant difference compared with 90° of flexion. In our study, a significantly higher score was present at 90° flexion compared with the 110° flexion in all groups.

There are several limitations of this study. The exact position of the brace was not additionally verified by a goniometer. This may have led to minimal changes in the results. Still, the published studies have shown displacement of less than 5° in each direction when verifying the position with a goniometer (8-10,14). Another limitation is that our maximum flexion angle during the testing was 120° due to the technical specifications of the elbow brace used, as well as the lack of assessment of supination and pronation angles. Vasen et al removed the anterior metal guard on the distal arm and proximal forearm to allow 135° elbow flexion, which was impossible with our brace (10). The patients in our study were all healthy, young volunteers, which may not represent the entire population undergoing elbow arthrodesis, limiting the generalizability of our results. Nevertheless, we believe that the tested mADL activities could become common ADLs of the general population over time, as young people get older. Additionally, these patients may benefit from physical or occupational therapy. Since the volunteers were healthy, this was not included in our study. Other studies suggest the use of adaptive aids, which may produce better results when examining different activities, such as using larger cutlery or glasses (10,14). Finally, the period of wearing the brace in a specific position was short, so longer use might lead to better results due to adaptation and compensatory movements of other joints. The same applies to the sequence of positions, which always started at 30° and finished at 120° of elbow flexion, allowing for adaptation during the examination process.

The optimal position of flexion for elbow arthrodesis is still a matter of debate and depends, among other factors, on the patient’s occupation and preference. Our study has shown favorable results for making elbow arthrodesis in 90° of flexion, which may also be applied to modern ADLs. However, patients should be consulted about the preferred angle for elbow arthrodesis, as no single fusion angle allows for the performance of all activities.
